# Hyaluronic Acid‐Decorated Liposomes for the Intrapulmonary Delivery of Imatinib: A Targeted Treatment for Postinflammatory Pulmonary Fibrosis

**DOI:** 10.1002/smsc.202500144

**Published:** 2025-06-10

**Authors:** Sara Bozzini, Valeria Bincoletto, Laura Pandolfi, Roberta Fusco, Rosanna Di Paola, Salvatore Cuzzocrea, Ilaria Andreana, Barbara Rolando, Eleonora Bozza, Cecilia Bagnera, Manuela Monti, Barbara Stella, Federica Meloni, Silvia Arpicco

**Affiliations:** ^1^ Second Department of Anesthesia and Intensive Care Fondazione IRCCS Policlinico San Matteo 27100 Pavia Italy; ^2^ Department of Drug Science and Technology University of Turin Via P. Giuria 9 10125 Turin Italy; ^3^ Laboratory of Respiratory Disease Section of Cell Biology UOS Transplant Center Fondazione IRCCS Policlinico San Matteo 27100 Pavia Italy; ^4^ Department of Chemical, Biological, Pharmaceutical and Environmental Sciences University of Messina 98166 Messina Italy; ^5^ Department of Veterinary Science University of Messina 98168 Messina Italy; ^6^ Department of Paediatric Oncoematology/Cell Factory Fondazione IRCCS Policlinico San Matteo 27100 Pavia Italy; ^7^ Research Center for Regenerative Medicine Fondazione IRCCS Policlinico San Matteo 27100 Pavia Italy; ^8^ Department of Cardio‐Thoracic, Vascular Sciences and Public Health University of Padua 35128 Padua Italy

**Keywords:** chronic lung allograft dysfunctions, imatinib, interstitial lung diseases, liposomes

## Abstract

Nanotechnology allows drugs to be delivered locally and specific cells to be targeted, leading to a promising new therapeutic approach for interstitial lung fibrosis. Hyaluronic acid (HA)‐decorated imatinib‐loaded liposomes (LIP‐HA44700‐Im) are developed to target CD44 positive cells for the inhalation treatment of fibrogenic lung disorders. LIP‐HA44700‐Im are assessed for their uptake and biological activity on respiratory effectors that are related to CD44 expression and compared to undecorated liposomes (LIP). LIP‐HA44700‐Im uptake is significantly higher than that of LIP, and most of the internalized LIP‐HA44700‐Im are colocalized with cellular endosomes. LIP‐HA44700‐Im also reduce lung fibroblasts viability. After 24 h, LIP‐HA44700‐Im are able to impair collagen 1a1 release and c‐Abl phosphorylation. Based on in vitro data, it has been assessed whether the intratracheal administration of LIP‐HA44700‐Im is able to prevent lung fibrosis in a mouse bleomycin model. The local administration of LIP‐HA44700‐Im is associated with a significant decrease in alveolar inflammation, lung fibrosis, collagen deposition, and TGF‐β expression. LIP‐HA44700‐Im target and deliver imatinib to lung pathogenic cells in vitro and represent a promising therapeutic option for the local treatment of fibrogenic lung disorders, although further development is required. These in vivo results confirm the validity of targeted nano‐based treatment for inflammatory‐driven lung fibrogenesis.

## Graphical abstract

Hyaluronic acid‐decorated imatinib‐loaded liposomes are developed to specific target CD44 receptor expressed by interstitial lung fibrosis cells, demonstrating higher uptake than empty liposomes. Based on promising in vitro data, they are administered intratracheally and intraperitoneally in a mouse bleomycin model, assessing their ability to decrease alveolar inflammation, lung fibrosis, collagen deposition, and TGF‐β expression.

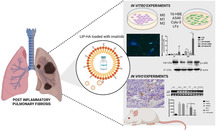

## 1 Introduction

Chronic fibrosis of the lung tissue and airways can occur as a consequence of intense and sustained inflammatory and immune (either auto‐ or allo‐immune reactions) triggers. The mechanisms of fibrogenesis have been partly elucidated; in most cases, alveolar walls are thickened by the proliferation of type 2 epithelial cells as well as by infiltration by inflammatory cells and fibroblasts, which differentiate into myofibroblasts, proliferate, and deposit extracellular matrix compounds. The spectrum of these diseases is very wide and includes several clinical pictures, which have been classified for clinical and prognostic purposes as follows: 1) secondary to a known disorder (such as sarcoidosis, connective tissue disorders, graft versus host disease, chronic lung rejection, and soon) or inhalation exposure to a certain agent (as for pneumoconiosis); 2) occurring without a specific cause (idiopathic). Although single disease entities are quite rare, fibrogenic lung disorders, taken together, account for significant morbidity with prevalence rates estimated to be around 74–76 cases per 100 000 people in Europe and USA.^[^ ^1^ ^]^ Chronic lung allograft dysfunction (CLAD), namely, chronic lung rejection^[^ ^2^ ^]^ and interstitial lung fibrosis associated with autoimmune diseases (collagen tissue disease‐interstitial lung diseases),^[^ ^3^ ^]^ are among the most typical forms of progressive lung fibrosis driven by allo‐ and autoimmune triggers.

CLAD is the main long‐term limitation to survival in lung transplant recipients,^[^ ^4^ ^]^ while autoimmune‐disease‐associated interstitial lung fibrosis represents more than 20% of all pulmonary fibrosis cases. In both settings, repeated inflammatory‐immune insults lead to epithelial mesenchymal transition (EMT) and the recruitment of tissue mesenchymal cells with the sustained proliferation of lung fibroblasts (LFs) and the accumulation of the extracellular matrix, in particular collagen deposition. The last two decades have witnessed a significant evolution in the treatment of pulmonary fibrosis with two oral antifibrotic drugs being licensed for the treatment of idiopathic fibrosis and progressive forms of secondary fibrosis, with several other drugs in the pipeline. However, the available approaches can, at best, slow disease progression with a significant, and sometimes limiting, degree of systemic toxicity. Research is therefore also focusing on new local (inhalation) treatment strategies, with many being based on nanotechnological approaches, whose investigation, in recent years, has led to the development of several nanocarriers of known and new drugs.^[^ ^5^, ^6^, ^7^, ^8^ ^]^ Encouraging results have also been reported for the intratracheal (IT) delivery of CD44‐targeted drug‐loaded gold nanoparticles (CD44Ab‐GNPs) in models of bleomycin‐induced lung fibrosis^[^ ^9^ ^]^ and heterotopic tracheal transplantation.^[^ ^10^ ^]^ Targeting of GNPs with an anti‐CD44 antibody directs the drug preferentially to LFs and macrophages, thus sparing, as much as possible, normal epithelial cells, which, as reported, do not express CD44 at a high rate.^[^ ^11^ ^]^ In these papers, imatinib (Im) was the drug of choice due to its well‐known chemical and toxicological profile and reported antifibrotic activity.^[^ ^12^, ^13^, ^14^, ^15^ ^]^ Its failure, when administered as an antifibrotic in a clinical trial on systemic sclerosis patients, was due to safety issues.^[^ ^16^ ^]^ However, while Im‐loaded CD44Ab‐GNPs have provided encouraging in vitro and in vivo results, these nanovectors have been found to accumulate and resist for long periods in alveolar macrophages, suggesting that unwanted toxic effects may occur with long‐term repeated use.^[^ ^17^ ^]^ Thus, using the higher biocompatibility profile of liposomes as a foundation, we have designed HA‐coated liposomes loaded with Im, with the aim of avoiding as much lung toxicity as possible, while increasing nanocarrier biocompatibility. Herein, we assess their in vitro and in vivo effects on a mouse model of bleomycin‐induced lung fibrosis after IT administration.

The proposed liposomes are mainly directed against highly CD44 positive cells thanks to surface modification with HA, with a molecular weight of 44 700 Da. HA is a physiological ligand of CD44 receptor, which is overexpressed by LFs and other mesenchymal cells, whereas it is not highly expressed by healthy, noninflamed, bronchial epithelium.^[^ ^11^ ^]^

## 2 Results

### 2.1 Liposomes Preparation and Characterization

Among the different promising drug delivery systems, such as for example extracellular vesicles,^[^ ^18^ ^]^ liposomes were chosen for Im delivery.

A range of different preparation methods and phospholipidic mixtures were tested to obtain suitable formulations for further investigation. Im mesylate, due to its hydrophilic nature, was encapsulated into the inner aqueous liposome (LIP‐Im) compartment using either the citrate or ammonium sulfate methods. Citrate buffer was selected to obtain a pH gradient‐mediated loading of Im that precipitates inside liposomes aqueous core as salt, as observed by the cryogenic‐transmission electron microscopy (cryo‐TEM) images (Figure S1, Supporting Information). Liposomes appeared unilamellar with a spherical shape, and drug precipitation inside their aqueous core was observed. Liposomes mean diameter was consistent with those measured by dynamic light scattering (**Table** **1**). The citrate method was selected because it showed the highest drug entrapment efficiency (EE%), the ratio between drug/lipid molar ratio after purification, and drug/lipid molar ratio after extrusion. The citrate method gave an EE of 97%, while ammonium sulfate provided a value of around 85%; this difference is due to differences in drug solubility, as Im solubility in citrate buffer (330 mg mL^−1^) is higher than in ammonium sulfate (280 mg mL^−1^).

**Table 1:** Physicochemical characteristics of liposomal formulations (means ± SD; *n* = 3).

Formulation	Mean diameter [nm] ± SD	PDI	Zeta potential [mV] ± SD	Entrapment efficiencya) [%] ± SD
LIP‐Im	212 ± 2	0.125	−31.3 ± 0.2	97 ± 3
LIP‐HA44700‐Im	262 ± 4	0.164	−28.0 ± 2.0	95 ± 3

a) Ratio between drug/lipid molar ratio after purification and drug/lipid molar ratio after extrusion.

The best formulation for LIP‐Im was found to be 70:30:3 molar ratio [1,2‐dipalmitoyl‐sn‐glycero‐3‐phosphocholine (DPPC):cholesterol (CHOL):L‐α‐phosphatidylglycerol (PG)] while, in the case of decorated LIP‐Im (LIP‐HA44700‐Im), PG was substituted with a conjugate between sodium hyaluronate (HA) (44 700 Da) and the 1,2‐dipalmitoyl‐sn‐glycero‐3‐phosphoethanolamine (DPPE) phospholipid (HA‐DPPE).^[^ ^19^ ^]^ LIP‐Im and LIP‐HA44700‐Im showed good and comparable drug EE%, indicating that the HA‐DPPE conjugate did not affect the drug encapsulation, as was demonstrated in a previous study.^[^ ^20^ ^]^ The physicochemical characteristics of the liposomal formulations are summarized in Table 1: LIP‐Im showed a mean diameter of around 210 nm, whereas there is an increase in LIP‐HA44700‐Im because of the presence of the HA‐DPPE conjugate. The phospholipid chains are incorporated into the hydrophobic bilayer, while HA is exposed to the aqueous phase. The polydispersion index (PDI) value was lower than 0.17 for all the formulations and the zeta potential value was around −30 mV.

**Figure 1:**
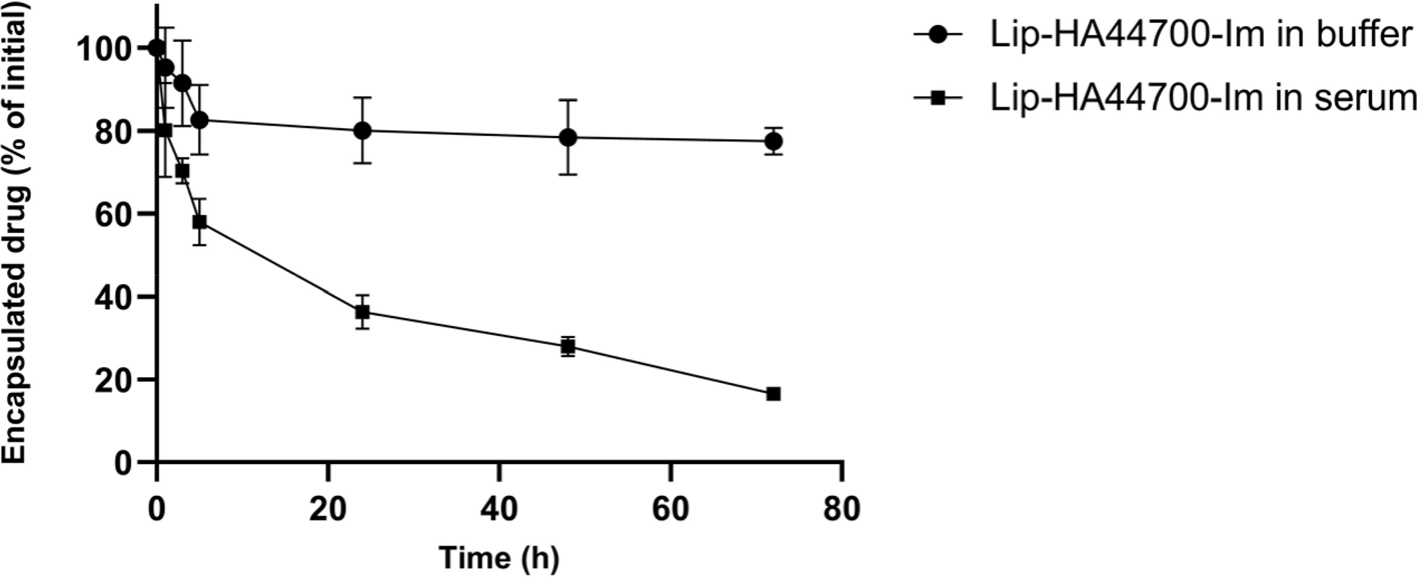
Im release from liposomes in HEPES buffer and FBS at 37 °C. Results are expressed as a percentage of the initial drug remaining in liposomes, at different periods, and are presented as mean ± SD (*n* = 3).

The release profile of LIP‐HA44700‐Im was evaluated in [4‐(2‐hydroxyethyl) piperazine‐1‐ethanesulfonic acid] (HEPES) buffer at 37 °C by testing drug and phospholipid content at different time points after purification by gel chromatography. LIP‐HA44700‐Im still encapsulated 80% of Im at 72 h. Drug release in fetal bovine serum (FBS) was also evaluated and Im content reached 20% after 72 h (**Figure** **1**). LIP‐Im showed a similar release profile in HEPES buffer and FBS at 37 °C, demonstrating that the HA‐DPPE conjugate did not affect the drug release profile.

After 3 weeks of storage in HEPES buffer at 4 °C, all formulations still retained 95% of the initial drug content, and no appreciable size and/or zeta potential changes were observed over this period.

### 2.2 Cell Internalization of Liposomes

**Figure 2:**
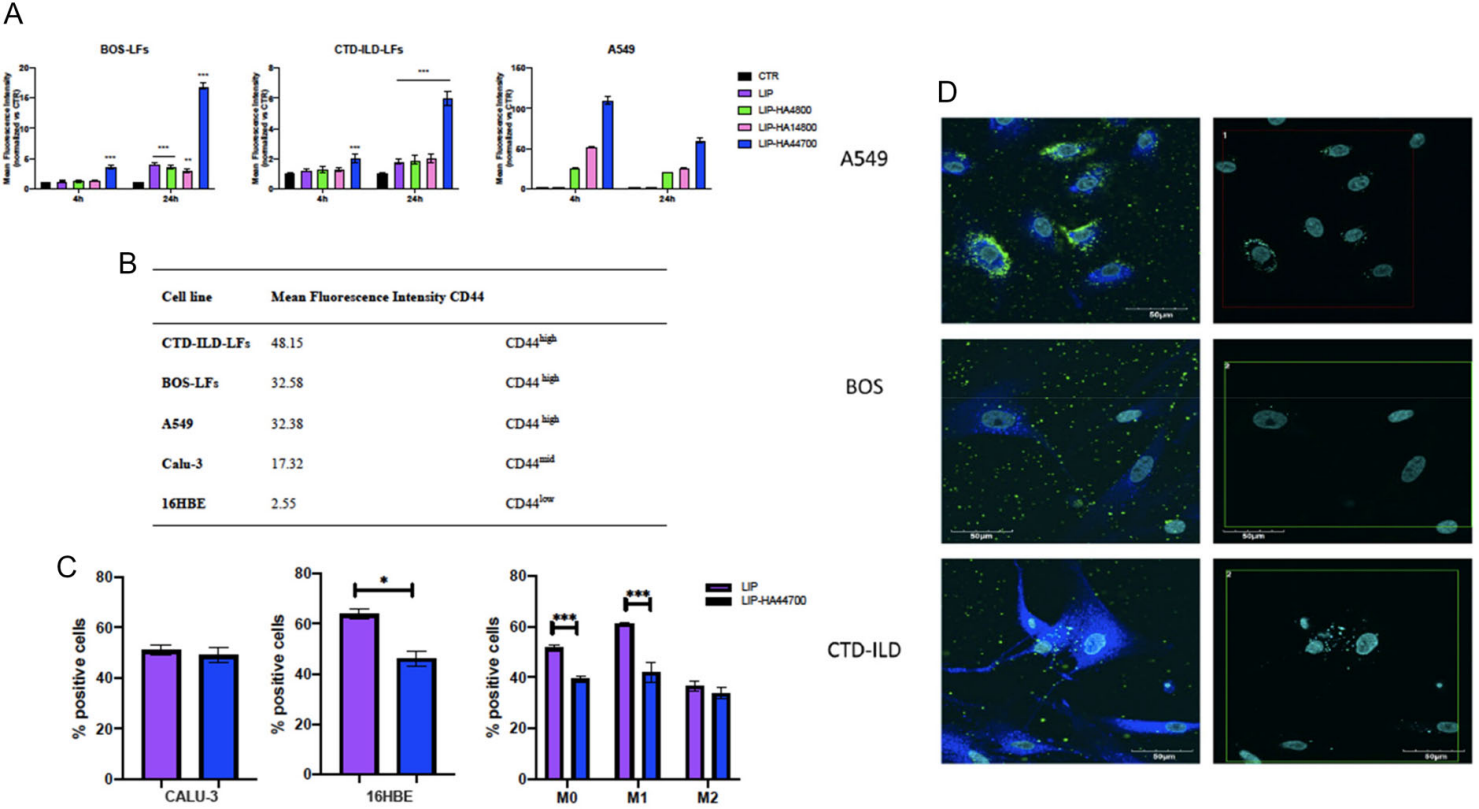
Liposome uptake analysis by flow cytometry and confocal microscopy: A) Flow cytometry analyses of fluorescently labeled LIP (purple), LIP‐HA4800 (green), LIP‐HA14800 (pink), and LIP‐HA44700 (blue) versus CTR (black) incubated with CD44 high BOS‐LFs, CTD‐ILD‐LFs, and A549 cells. Histograms represent the means of mean fluorescence intensity of liposomes ± standard deviation (SD). *n* of repetitions = 3, Student's *t*‐test was used for comparisons between groups. *p < 0.05 versus CTR; **p < 0.01 versus CTR; ***p < 0.001. B) Rate of CD44 expression of CTD‐ILD‐LFs, BOS‐LFs, A549, Calu‐3, and 16HBE obtained by flow cytometry analyses. C) Flow cytometry analysis of fluorescently labeled LIP, LIP‐HA44700 incubated for 4 h with CD44^low^ cells Calu‐3 and 16HBE epithelial cells and macrophages. Histograms represent the means of % positive cells ± SD. *n* of repetitions = 3, Student's *t*‐test was used for comparisons between groups. ***p < 0.001; **p < 0.005; *p < 0.05. D) Confocal images of LIP‐HA44700, fluorescently labeled and incubated with A549, BOS‐LFs, and CTD‐ILD‐LFs. Nuclei of cells = light blue (DAPI); liposomes = green signals; endosomes = blue signals. Scale bar = 50 μm.

To study the interactions between the liposomal preparations and bronchiolitis obliterans syndrome (BOS)‐derived LFs (BOS‐LFs), connective tissue disease‐associated interstitial lung disease (CTD‐ILD)‐derived LFs (CTD‐ILD‐LFs) and A549 cells, liposome internalization was analyzed using flow cytometry. We preliminarily tested fluorescently labeled liposomes decorated with HA of different molecular weights (MW) and demonstrated that the degree of cellular uptake was directly related to HA MW in all cell lines. In the experiments, liposomes were prepared with conjugates that had been previously synthetized in our laboratory.^[^ ^19^ ^]^ As expected, we observed in accordance with CD44‐expression, higher interactions between decorated liposomes (LIP‐HA44700) and BOS‐LFs, CTD‐ILD‐LFs and A549, compared to undecorated liposomes (LIP) (**Figure** **2**A).

The rates of CD44 expression of CTD‐ILD‐LFs, BOS‐LFs, A549, Calu‐3, and 16HBE are reported in Figure 2B, which shows that cells recovered from interstitial lung disease (ILD) cells and A549 cells, a type II alveolar epithelial cell line derived from lung adenocarcinoma, express CD44 at the highest rates.^[^ ^21^, ^22^ ^]^

We also assessed the uptake of LIP‐HA44700 by the CD44^mid^ epithelial cell line (Calu‐3), CD44^low^ epithelial cell line 16HBE, and macrophages derived from THP‐1 cells with specific differentiation toward in M0/M1/M2 phenotypes (Figure 2C). As expected, due to the lower expression rate of CD44, liposome uptake in epithelial cells was more “aspecific” as demonstrated by the uptake reduced internalization rate of LIP‐HA44700 compared to LIP. As for macrophages, again “aspecific” LIP uptake was high in M0 and M1 with significantly lower rates after 4 h incubations with LIP‐HA44700. In the case of M2 macrophages, lower rates of uptake were detected without any significant variation between LIP and LIP‐HA44700.

Data on uptake were also confirmed by confocal microscopy and show that most of the internalized liposomes in CD44^high^ cells colocalize with cellular endosomes after 24 h of incubation. Figure 2D shows that mainly LIP‐HA44700 was internalized by A549 cells, BOS‐LFs and CTD‐ILD‐LFs.

### 2.3 Cell‐Viability Assay

The uptake experiments demonstrated that higher HA MW resulted in higher uptake efficiency and that HA functionalization led to higher uptake by fibrogenic effectors and lower uptake by CD44^low^ epithelial cell lines and macrophages. It was therefore decided that LIP‐HA44700‐Im was to be further developed and assessed.

**Figure 3:**
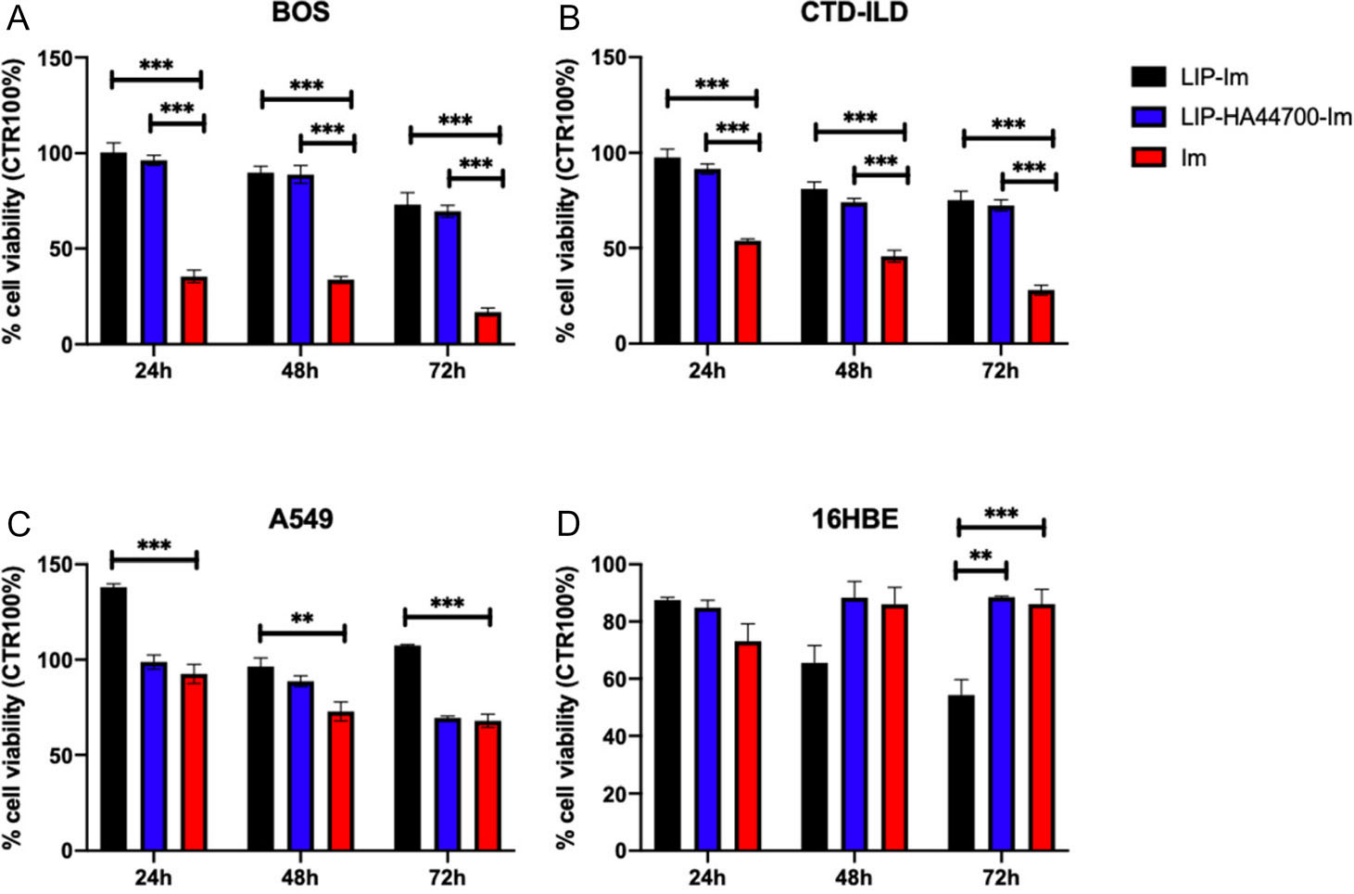
Viability, by MTT test, of A) BOS‐LFs, B) CTD‐ILD‐LFs, C) A549, and D) 16HBE cells after Im, LIP‐Im, and LIP‐HA44700‐Im treatment. The results are shown as mean ± SD of values from three independent experiments after 24–48–72 h of incubation. Student's *t*‐test was used for comparisons between groups. **p < 0.005; ***p < 0.001.

In order to assess the in vitro effects of these formulations, we decided to perform viability/cytotoxicity tests (by MTT—(3‐(4,5‐dimethylthiazol‐2‐yl)‐2,5‐diphenyltetrazolium bromide)‐/LDH—lactate dehydrogenase‐release assays), and compare the results with those of Im at the same concentration (30 μM). LIP‐Im and LIP‐HA44700‐Im reduced the viability of LFs by 30%, while free Im reduced the viability of the LFs by 80% (**Figure** **3**).

For epithelial cells, we observed: 1) a reduction in cell viability of 30% at 72 h after the treatment of A549 CD44^high^ cells with Im and LIP‐HA44700‐Im. LIP‐Im did not significantly affect cell viability; 2) a significant reduction in 16HBE cell viability 72 h after treatment with LIP‐Im (45.7% with LIP‐Im vs 11.5% with LIP‐HA44700‐Im and 13.9% with Im).

**Figure 4:**
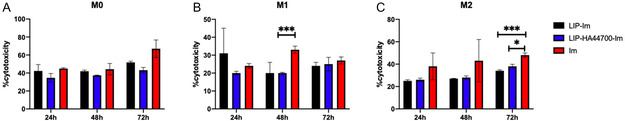
Cytotoxicity on A) M0, B) M1, and C) M2 macrophages after treatment with LIP‐Im, LIP‐HA44700‐Im, and Im. Cytotoxicity was assessed by LDH activity, detected using CytoTox 96 Non‐Radioactive Cytotoxicity Assay. The results are shown as mean ± SD of values from three independent experiments after 24–48–72 h of incubation. Student's *t*‐test was used for comparisons between groups. *p < 0.05; ***p < 0.001.

Macrophage cytotoxicity was also assessed using an LDH‐release assay (**Figure** **4**), which showed that LIP‐Im, LIP‐HA44700‐Im, and Im induced a significant degree of cytotoxicity (36–63% within 72 h of treatment on M0 macrophages without any significant variation among treatments) (Figure 4A). While lower rates of cytotoxicity were detectable in M1 cells with all preparations, but after 48 h, a significantly higher effect was detectable with Im compared to LIP‐HA44700‐Im (Figure 4B). Finally, Im elicited higher LDH release in M2 macrophages compared to LIP‐Im and LIP‐HA44700‐Im (Figure 4C).

#### 2.3.1 Effect of Liposomes on cAbl and Collagen Production by LFs

**Figure 5:**
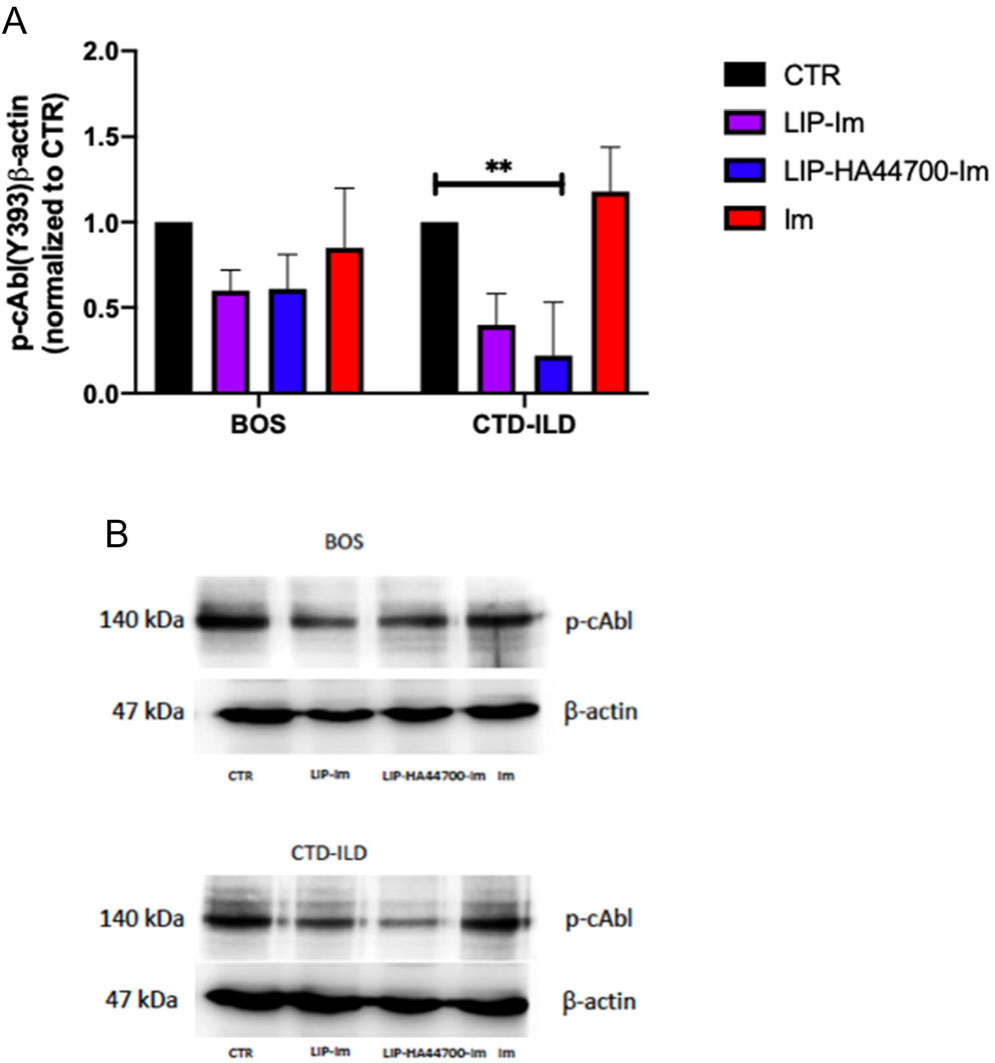
Western blot analysis of p‐cAbl in LFs derived from BOS and CTD‐ILD after 24 h of treatment with liposome and Im alone. A) Quantitative analysis of immunoblots representing the expression level of p‐cAbl in BOS and CTD‐ILD normalized to CTR = 1. B) Representative blot of immunedecoration using anti‐p‐cAbl and β‐actin. *n* = 3, Student's *t*‐test was used for comparisons between groups. **p < 0.005.

Knowing that cAbl is a specific target of Im, we decided to evaluate the effectiveness of LIP‐HA44700‐Im in inhibiting the activity of cAbl on pathogenic target cells: BOS and CTD‐ILD LFs. In order to study the activity of cAbl, the level of phosphorylation of the protein was evaluated. Western blot analysis showed, in LFs derived from patients with BOS, a 50% reduction in the activity of cAbl with both LIP‐Im and LIP‐HA44700‐Im, after 24 h of treatment. When analyzing the LFs derived from CTD‐ILD, differences in the activity of LIP‐Im and LIP‐HA44700‐Im were observed; LIP‐Im reduces the activity of cAbl by 60%, while LIP‐HA44700‐Im reduces it by 70%. Interestingly, Im has no effect, over 24 h, on the activity of cAbl in cells derived from BOS and CTD‐ILD (**Figure** **5**).

**Figure 6:**
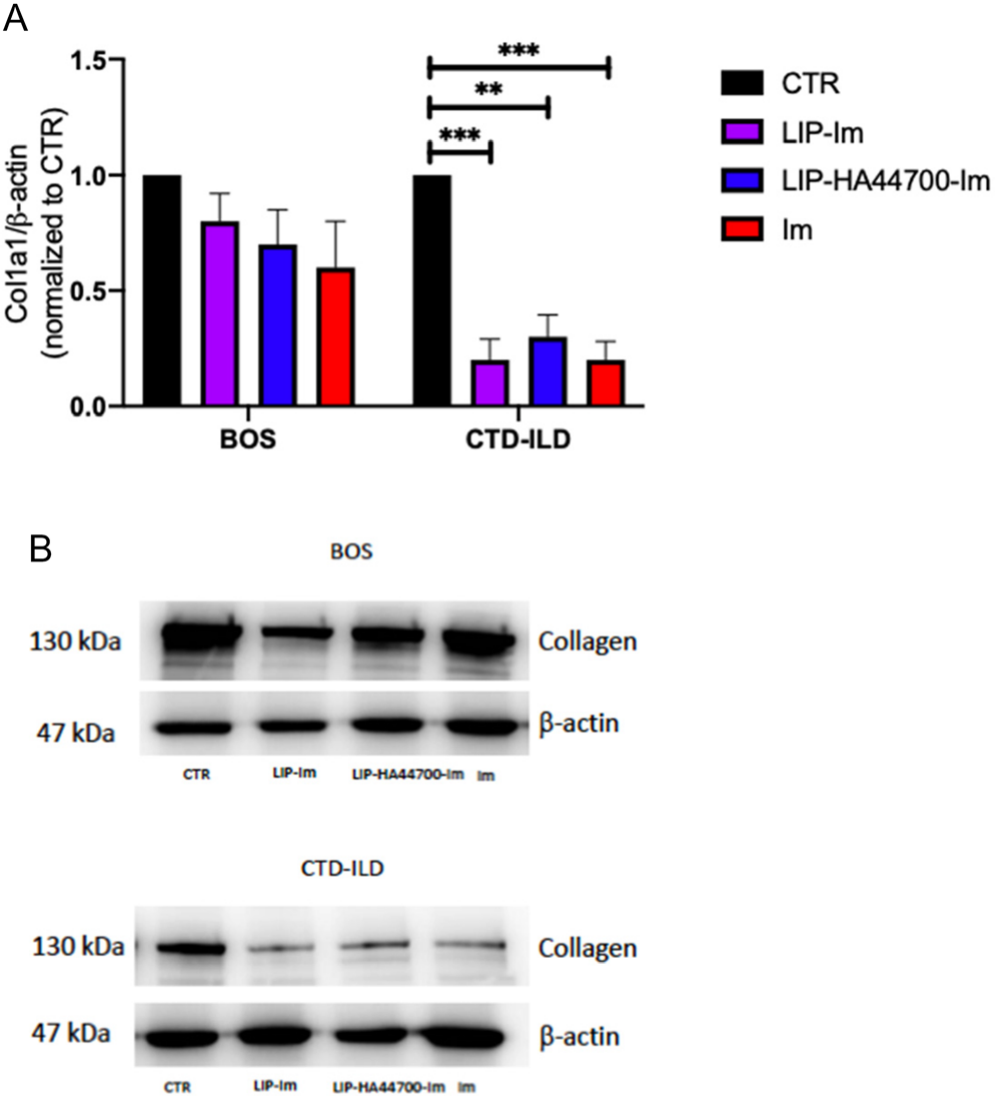
Western blot analysis of type 1a1 collagen in LFs derived from BOS and CTD‐ILD after 24 h of treatment with liposome and Im alone. A) Quantitative analysis of immunoblots representing the expression level of Col1a1 in BOS and CTD‐ILD. B) Representative blot of immunedecoration using anti Col1a1 and anti β‐actin. *n* = 3, Student's *t*‐test was used for comparisons between groups. **p < 0.005; ***p < 0.001.

Finally, the production of type 1 collagen, a key factor in the extracellular matrix that makes up fibrotic lesions, was evaluated by western blot analysis after 24 h of treatment with LIP‐Im, LIP‐HA44700‐Im and Im. It was found that LIP‐HA44700‐Im reduces the production of collagen by 30%, in LFs derived from BOS, while LIP‐Im reduces it by 20%. A 60% reduction in collagen expression was observed in LFs derived from CTD‐ILD after treatment with LIP‐Im and LIP‐HA44700‐Im. Im showed activity in reducing collagen expression by 50% in BOS‐LFs and by 60% in CTD‐ILD‐LFs (**Figure** **6**).

#### 2.3.2 Effect of Liposomes on THP‐1‐Derived Macrophages

**Figure 7:**
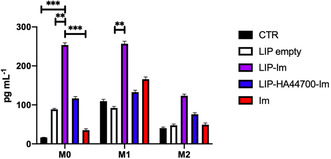
Effect of liposomes on THP‐1 derived macrophages. IL‐6 concentrations are expressed in pg mL^−1^. Results are shown as mean ± SD of values from three determinations. Student's *t*‐test was used for comparisons between groups. **p < 0.005; ***p < 0.001.

With the aim of determining the effect of liposomes, in terms of inflammatory activation, on macrophages, we measured the levels of Interleukin‐6 (IL‐6) released by THP‐1‐derived macrophages (M0, M1, and M2) after 48 h of incubation with LIP, LIP‐HA44700‐Im, LIP‐Im, and Im. As can be seen in **Figure** **7**, liposomes were able to activate macrophages and stimulate the release of IL‐6, even in the absence of the drug (LIP), above the levels observed in the untreated cells (CTR). Similar activity was observed with LIP‐HA44700‐Im, while a significantly greater stimulatory effect was observed when macrophages were cultured in the presence of LIP‐Im. These results are similar for all three macrophage phenotypes considered.

### 2.4 Effect of Liposomes on Mortality and Weight Loss in Bleomycin‐Induced Mice‐Lung Fibrosis

**Figure 8:**
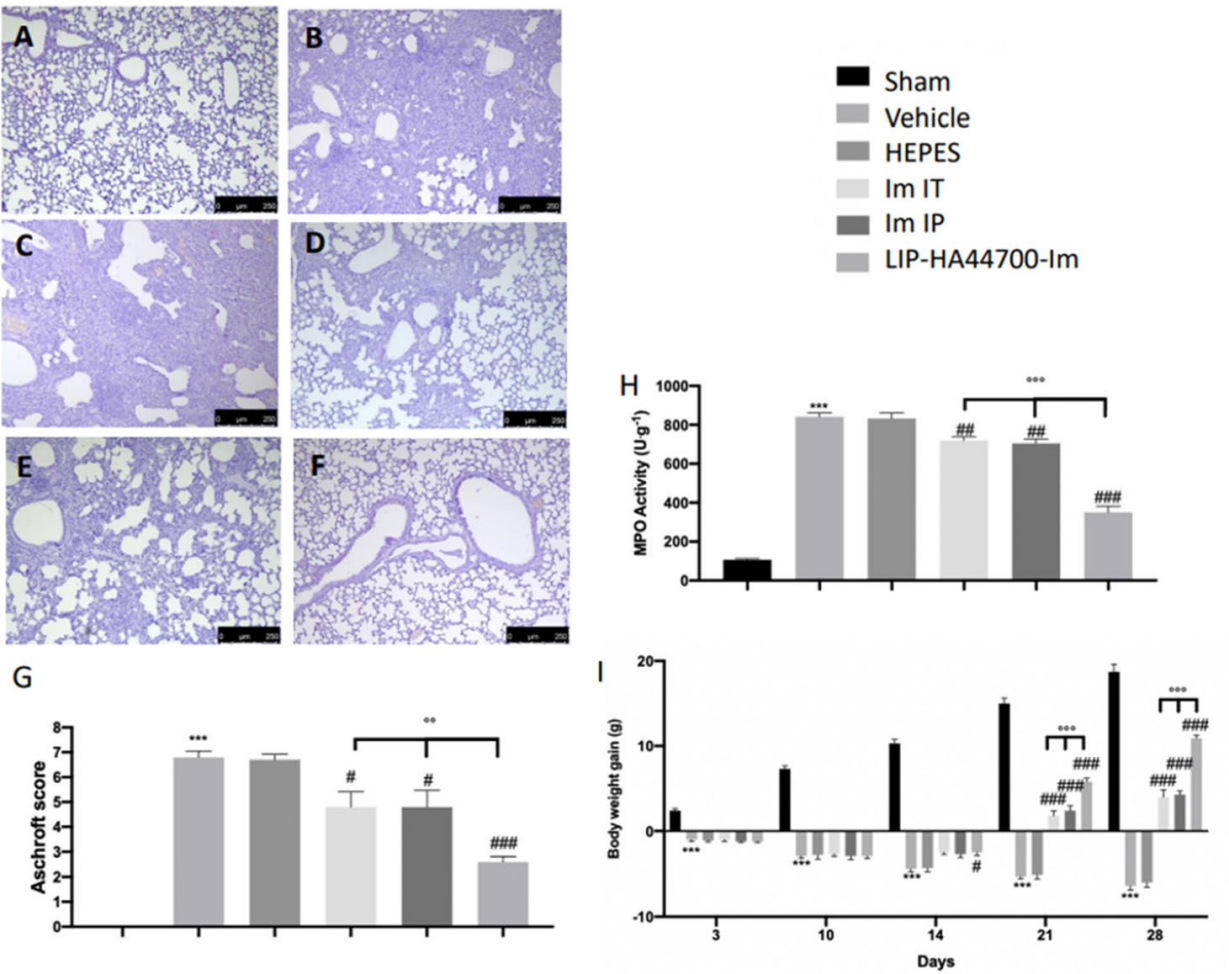
Effect of LIP‐HA44700‐Im on histological changes, MPO activity, and body weight. Hematoxylin and eosin staining of A) sham, B) vehicle, C) HEPES, D) Im‐IT, E) Im‐IP, F) LIP‐HA44700‐Im, G) histological injury score, H) MPO activity, and I) body weight gain. Figures are expressed as the mean ± standard error of the mean (SEM) of *n* = 10 number of animals. The results were analyzed by one‐ or two‐way ANOVA followed by a Bonferroni post hoc or Dunnett post hoc test for multiple comparisons. A *p*‐value <0.05 was considered significant. *p < 0.05 versus sham, #*p* < 0.05 versus vehicle, **p < 0.01 versus sham, ##*p* < 0.01 versus vehicle, ***p < 0.001 versus sham, ###*p* < 0.001 versus vehicle, °*p* < 0.05 versus Im‐IT and Im‐IP, °°*p* < 0.01 versus Im‐IT and Im‐IP, °°°*p* < 0.001 versus Im‐IT and Im‐IP.

The encouraging in vitro data showed that HA44700 functionalization grants LIP‐Im improved targeting and higher c‐Abl inhibition toward pathogenic LFs, lower aspecific uptake and cytotoxicity in CD44^low^ 16HBE epithelial cells and a trend toward lower proinflammatory activity in macrophage lines. Animal experiments were therefore designed to assess the efficacy of locally delivered LIP‐HA44700‐Im on a model of postinflammatory lung fibrosis. The efficacy of LIP‐HA44700‐Im was compared with that of the Im free drug, with both being delivered either by IT or intraperitoneal (IP) route, at the same concentration. Twenty‐eight days after bleomycin instillation, the histological analysis of lung samples harvested from vehicle‐treated mice (**Figure** **8**B,G) and HEPES‐treated mice (Figure 8C,G) showed higher tissue injury and extracellular matrix deposition, compared to the sham animals (Figure 8A,G). Im‐IT, Im‐IP, and LIP‐HA44700‐Im‐IT treatment reduced lung damage and pulmonary edema, as shown by hematoxylin and eosin staining. In particular, LIP‐HA44700‐Im treatment was significantly more effective than Im‐IT and Im‐IP (Figure 8D–G) treatments. LIP‐HA44700‐Im was able to reduce neutrophil activity, as shown by an myeloperossidase (MPO) assay (Figure 8H) and pulmonary‐fibrosis‐associated body weight loss (Figure 8I).

### 2.5 Effect of Liposomes on Bleomycin‐Induced T‐Lymphocyte Infiltration

**Figure 9:**
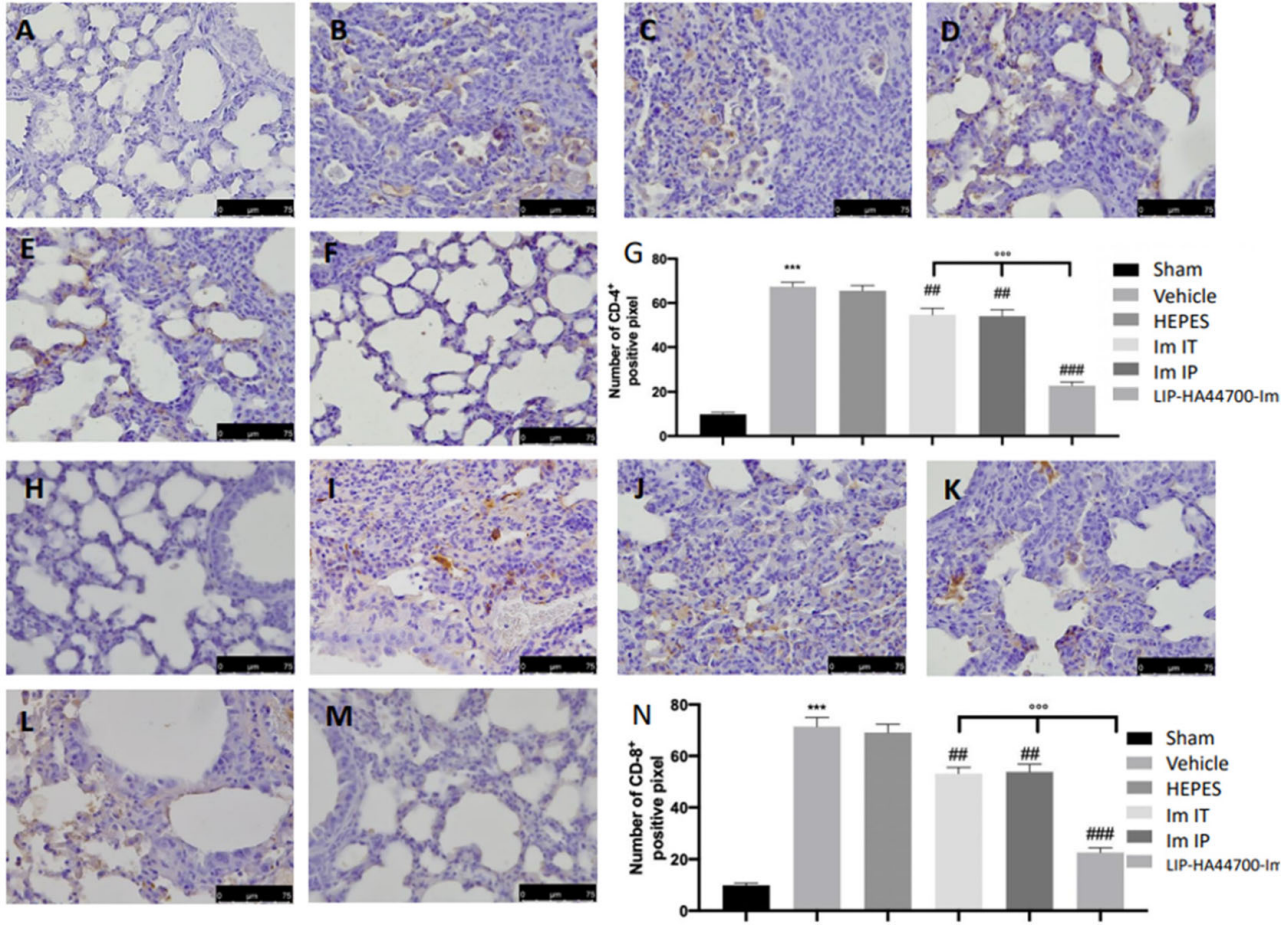
Effect of LIP‐HA44700‐Im on CD4 and CD8 expression. Immunohistochemical analysis of CD4: A) sham, B) vehicle, C) HEPES, D) Im‐IT, E) Im‐IP, F) LIP‐HA44700‐Im, G) number of positive cells/field, immunohistochemical analysis of CD8: H) sham, I) vehicle, J) HEPES, K) Im‐IT, L) Im‐IP, M) LIP‐HA44700‐Im, and N) number of positive cells/field. Figures are expressed as the mean ± SEM of *n* = 10 number of animals. The results were analyzed by one‐way ANOVA followed by a Bonferroni post hoc or Dunnett post hoc test for multiple comparisons. A *p*‐value < 0.05 was considered significant. *p < 0.05 versus sham, #*p* < 0.05 versus vehicle, **p < 0.01 versus sham, ##*p* < 0.01 versus vehicle, ***p < 0.001 versus sham, ###*p* < 0.001 versus vehicle, °*p* < 0.05 versus Im‐IT and Im‐IP, °°*p* < 0.01 versus Im‐IT and Im‐IP, °°°*p* < 0.001 versus Im‐IT and Im‐IP.

Twenty‐eight days after bleomycin instillation, vehicle‐ and HEPES‐treated animals showed an increased number of CD4 and CD8 positive cells (**Figure** **9**B,C,G,J,N), compared to sham animals (Figure 9AH). Im‐IT, Im‐IP, and LIP‐HA44700‐Im treatment reduced these numbers (Figure 9D–F,K–M). In particular, LIP‐HA44700‐Im treatment was significantly more effective than the Im‐IT and Im‐IP treatments.

### 2.6 Effect of Liposomes on Cell Infiltration

**Figure 10:**
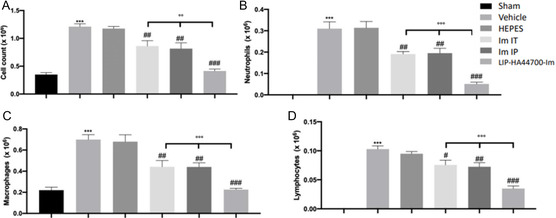
Effect of LIP‐HA44700‐Im on bronchoalveolar lavage (BAL) analysis. A) Total cell count, B) neutrophils, C) macrophages, and D) lymphocytes. All values in the figures and text are expressed as the mean ± SEM of *n* number of animals. Figures are expressed as the mean ± SEM of *n* = 10 number of animals. The results were analyzed by one‐way ANOVA followed by a Bonferroni post hoc or Dunnett post hoc test for multiple comparisons. A *p*‐value < 0.05 was considered significant. *p < 0.05 versus sham, #*p* < 0.05 versus vehicle, **p < 0.01 versus sham, ##*p* < 0.01 versus vehicle, ***p < 0.001 versus sham, ###*p* < 0.001 versus vehicle, °*p* < 0.05 versus Im‐IT and Im‐IP, °°*p* < 0.01 versus Im‐IT and Im‐IP, °°°*p* < 0.001 versus Im‐IT and Im‐IP.

Twenty‐eight days after bleomycin instillation, vehicle‐ and HEPES‐treated mice showed increased inflammatory‐cell recruitment in the bronchoalveolar lavage fluid (BALf), compared to the sham group (**Figure** **10**A). Specifically, we evaluated neutrophils (Figure 10B), macrophages (Figure 1C) and lymphocytes (Figure 10D), and detected a significant increase in cell numbers. LIP‐HA44700‐Im significantly reduced the inflammatory‐cell infiltration in the BALf.

### 2.7 Effect of Liposomes on Bleomycin‐Induced Lung Fibrotic Changes

**Figure 11:**
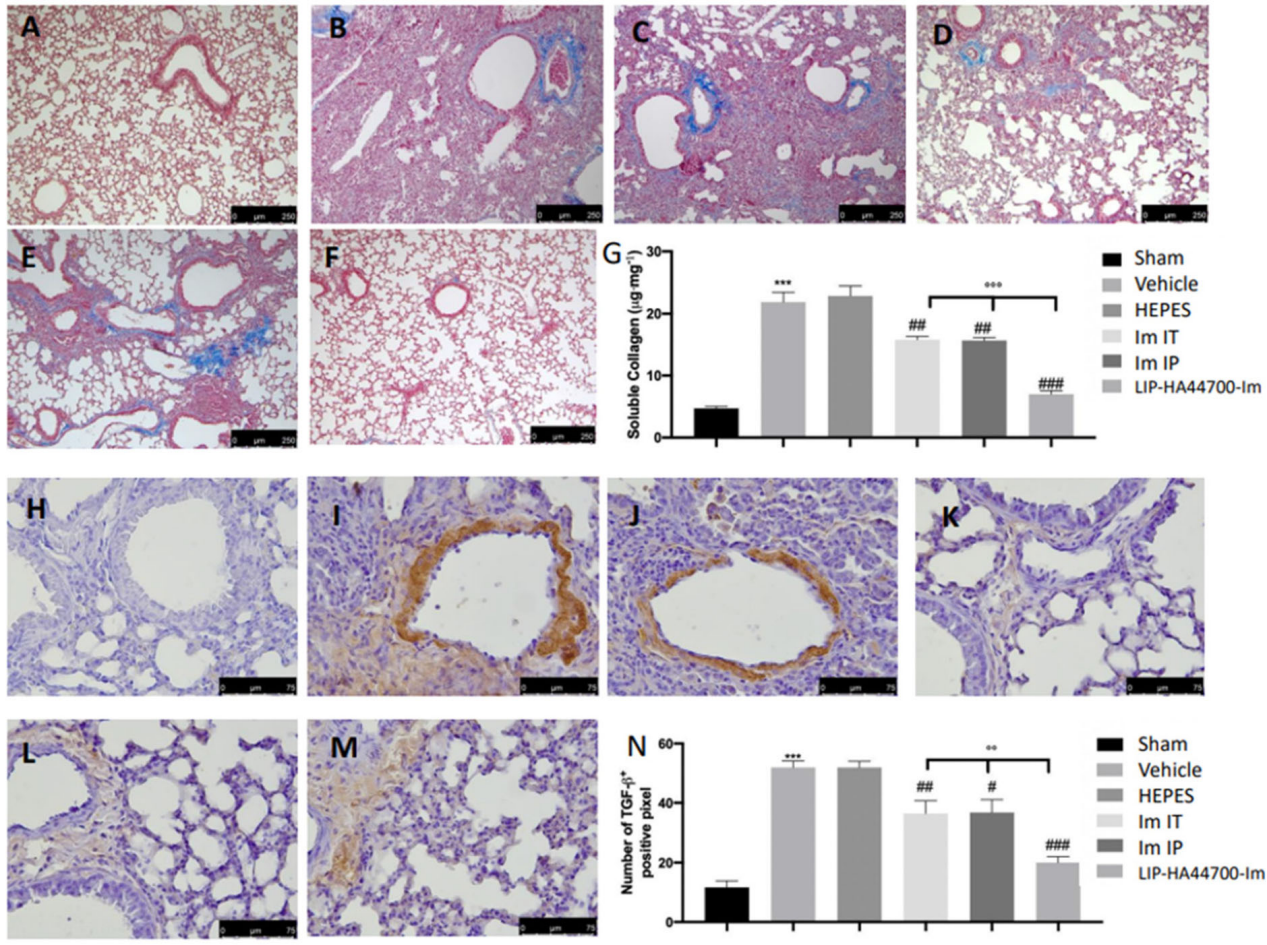
Effect of LIP‐HA44700‐Im on collagen deposition and TGF‐β expression. Masson trichrome staining: A) sham, B) vehicle, C) HEPES, D) Im‐IT, E) Im‐IP, F) LIP‐HA44700‐Im, G) sircol soluble collagen assay; immunohistochemical analysis of TGF‐1β: H) sham, I) vehicle, J) HEPES, K) Im‐IT, L) Im‐IP, M) LIP‐HA44700‐Im, and N) number of positive cells/field. Figures are expressed as the mean ± SEM of *n* = 10 number of animals. The results were analyzed by one‐way ANOVA followed by a Bonferroni post hoc or Dunnett post hoc test for multiple comparisons. A *p*‐value < 0.05 was considered significant. *p < 0.05 versus sham, #*p* < 0.05 versus vehicle, **p < 0.01 versus sham, ##*p* < 0.01 versus vehicle, ***p < 0.001 versus sham, ###*p* < 0.001 versus vehicle, °*p* < 0.05 versus Im‐T and Im‐IP, °°*p* < 0.01 versus Im‐IT and Im‐IP, °°°*p* < 0.001 versus Im‐IT and Im‐IP.

Masson trichrome staining showed increased blue areas in tissues from vehicles and HEPES‐treated mice, compared to sham tissue (**Figure** **11**A–C,G). Im‐IT, Im‐IP, and LIP‐HA44700‐Im (Figure 11D–G) treatment reduced collagen deposition. In order to evaluate the mechanisms of fibrogenesis, we also performed immunohistochemical analyses for TGF‐β expression. No positive staining was detected in the sham group (Figure 11H,N). Vehicle‐ and HEPES‐treated mice showed increased TGF‐β expression, while Im‐IT, Im‐IP and LIP‐HA44700‐Im (Figure 11I–N) treatment reduced TGF‐β immunostaining. In particular, LIP‐HA44700‐Im (Figure 11F,G,M,N) treatment was significantly more effective than Im‐IT.

### 2.8 Effect of Liposomes on MAPK and NF‐kB

**Figure 12:**
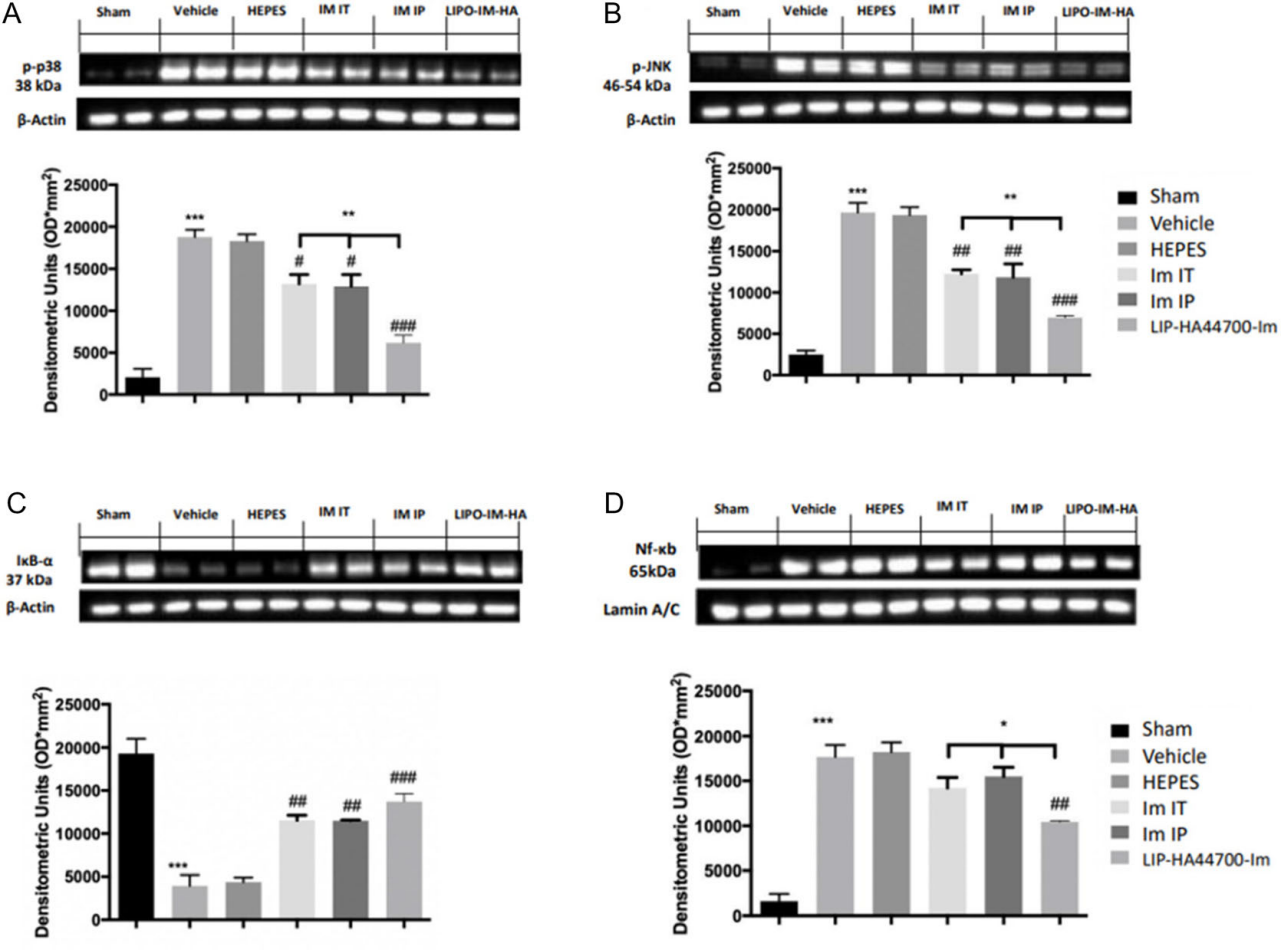
Effect of LIP‐HA44700‐Im on MAPK and NF‐kB pathways. Western blot analysis of: A) p‐p38, B) p‐JNK, C) IkB‐apha, and D) NF‐kB D). Figures are expressed as the mean ± SEM of *n* = 10 number of animals. The results were analyzed by one‐way ANOVA followed by a Bonferroni post hoc or Dunnett post hoc test for multiple comparisons. A *p*‐value < 0.05 was considered significant. *p < 0.05 versus sham, #*p* < 0.05 versus vehicle, **p < 0.01 versus sham, ##*p* < 0.01 versus vehicle, ***p < 0.001 versus sham, ###*p* < 0.001 versus vehicle, °*p* < 0.05 versus Im‐IT and Im‐IP, °°*p* < 0.01 versus Im‐IT and Im‐IP, °°°*p* < 0.001 versus Im‐IT and Im‐IP.

Twenty‐eight days after bleomycin installation, vehicle‐ and HEPES‐treated animals showed increased p‐p38 and p‐JNK expressions, as compared to sham animals. Im‐IT, Im‐IP, and LIP‐HA44700‐Im treatment reduced p‐p38 and p‐JNK expressions (**Figure** **12**A,B).

Western blot analysis showed that vehicle and HEPES‐treated animals displayed reduced IkB‐alpha expression in the cytoplasm and increased nuclear NF‐kB expression, compared to sham animals. Im‐IT, Im‐IP, and LIP‐HA44700‐Im treatment increased cytosolic IkB‐alpha expression (Figure 12C) and reduced nuclear NF‐kB expression (Figure 12D). In particular, LIP‐HA44700‐Im treatment was significantly more effective than Im‐IT and Im‐IP treatments (Figure 12A–D).

### 2.9 Effect of Liposomes on MMPs and TRAF‐6 Expression

**Figure 13:**
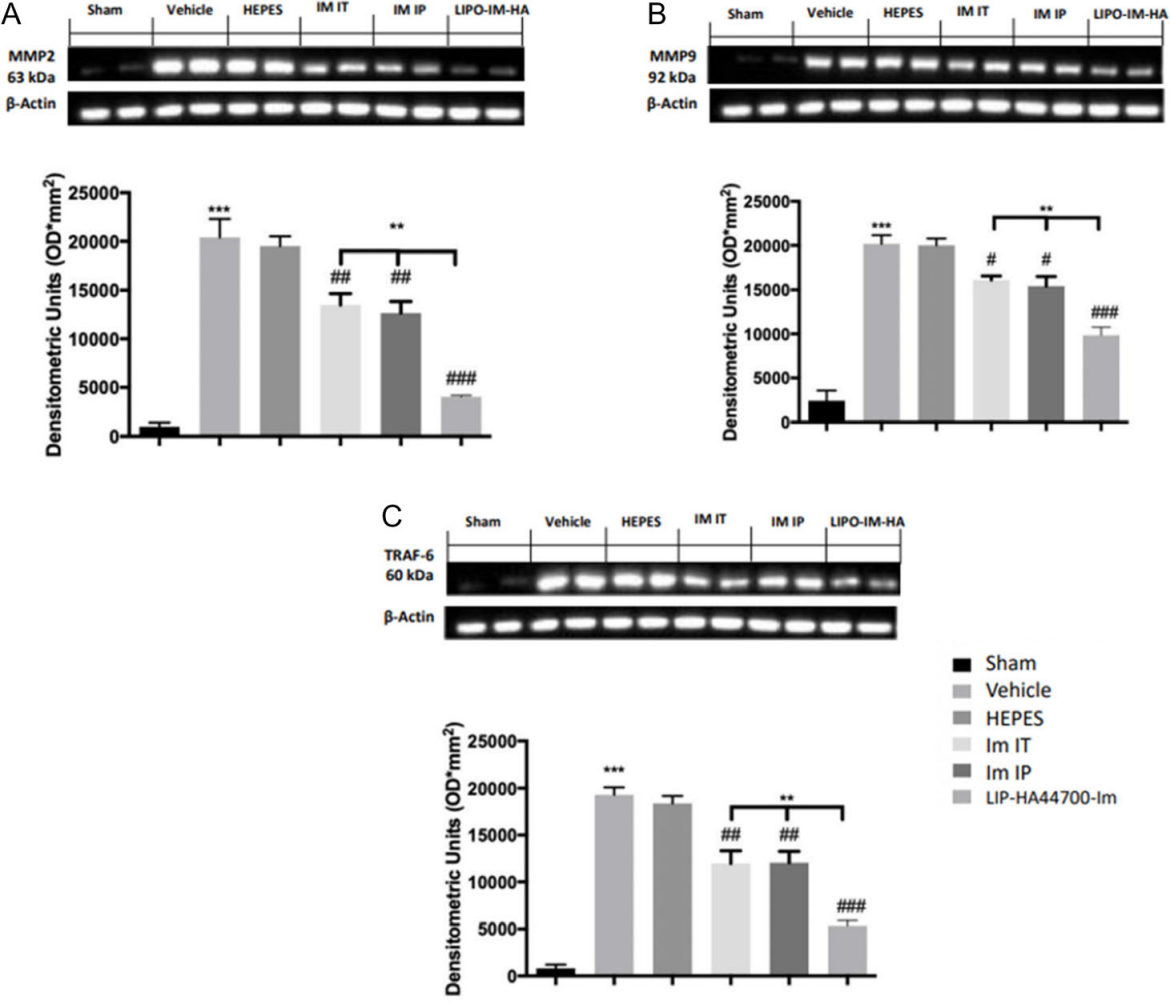
Effect of LIP‐HA44700‐Im on MMPs and TRAF‐6 pathways. Western blot analysis of: A) MMP2, B) MMP9, and C) TRAF‐6. Figures are expressed as the mean ± SEM of *n* = 10 number of animals. The results were analyzed by one‐way ANOVA followed by a Bonferroni post hoc or Dunnett post hoc test for multiple comparisons. A *p*‐value < 0.05 was considered significant. *p < 0.05 versus sham, #*p* < 0.05 versus vehicle, **p < 0.01 versus sham, ##*p *< 0.01 versus vehicle, ***p < 0.001 versus sham, ###*p* < 0.001 versus vehicle, °*p* < 0.05 versus Im‐IT and Im‐IP, °°*p* < 0.01 versus Im‐IT and Im‐IP, °°°*p* < 0.001 versus Im‐IT and Im‐IP.

Twenty‐eight days after bleomycin instillation, vehicle‐ and HEPES‐treated animals showed increased MMP‐2, MMP‐9, and TRAF‐6 expression, compared to sham animals (**Figure** **13**A–C). Im‐IT, Im‐IP, and LIP‐HA44700‐Im treated animals showed reduced MMP‐2, MMP‐9, and TRAF‐6 expression. LIP‐HA44700‐Im treatment was significantly more effective than Im‐IT and Im‐IP treatment (Figure 13A–C).

## 3 Discussion

The results of the study demonstrate the in vitro and in vivo efficacy of the actively targeted liposomal Im formulation, which is designed to be delivered via the local route. Thanks to the positive experience that our group has had working with another nanovehicle, anti‐CD44 coated Im‐loaded GNPs,^[^ ^9^, ^10^ ^]^ we have now obtained a more biocompatible and easy‐to‐prepare nanocarrier. The aim here is to directly deliver high drug doses to inflammatory effectors, in particular alveolar macrophages, and spare CD44 negative bronchial epithelial cells as much as possible. This would also allow for sustained intracellular drug release over time and the lowest possible level of systemic and local toxicity. Active targeting has been achieved by decorating liposomes with a conjugate obtained by covalently linking HA (44 700 Da) with phospholipids, which are the major components of liposomes. By adding a 3% molar ratio of conjugate to the liposomal formulation, we have obtained a stable suspension that is able to efficiently recognize and interact with the CD44 receptor. This approach is highly innovative and promising as it is the first targeted approach, to the best of our knowledge, which has been designed and developed for the inhalation treatment of chronic respiratory disorders. The literature shows that previous Im‐ and HA‐containing liposomal formulations were either obtained by adsorbing the negatively charged HA onto the surface of cationic liposomes^[^ ^23^, ^24^ ^]^ or, in a recent paper, by conjugating HA to DSPE‐PEG2000‐NH_2_ through an amide bond.^[^ ^25^ ^]^ Liposomes proposed for inhalation have been made of neutral phospholipids^[^ ^26^ ^]^ and, in particular, the liposomal amikacin formulation for inhalation (Arikayce) is composed of DPPC and CHOL, which was very similar to pulmonary surfactant.^[^ ^27^ ^]^ Starting from these considerations, DPPC was chosen as the principal phospholipid component of our liposomes, which were designed to encapsulate Im via insertion into the aqueous core using the active drug encapsulation technique with either the citrate or ammonium sulfate methods. Differences in the amount of encapsulated drug were observed. The highest drug entrapment efficiency, around 97%, was obtained using citrate, whereas ammonium sulfate gave 85%, with this disparity being caused by differences in Im solubility in the two media. Our encapsulation efficiency results were higher or comparable to those reported in the literature, where different phospholipid compositions were used.^[^ ^28^ ^]^

The liposomal formulations described in the literature were obtained using the thin‐lipid‐film hydration method to encapsulate Im,^[^ ^29^, ^30^ ^]^ ethanol injection,^[^ ^25^ ^]^ and the solvent‐evaporation method to encapsulate the Im sodium deoxycholate complex.^[^ ^23^, ^24^ ^]^

Preliminary in vitro experiments were performed to determine the HA MW that would provide the highest CD44 positive cells targeting efficiency. In fact, LIP‐HA44700 uptake by CD44^high^ LFs and by CD44^low^ 16HBE was significantly different from that of LIP. However, a rate of “aspecific” liposome uptake was present in all cell types, unlike previous observations with CD44‐coated GNPs.^[^ ^9^, ^17^ ^]^

Im loading was also performed using an innovative strategy, which provides the drug with high stability in the aqueous core and slows down drug release over time. A single‐shot treatment of LFs with LIP‐HA44700‐Im was able to induce significant inhibition in c‐Abl phosphorylation and the production of collagen1 by LFs, and to reduce the proinflammatory activity of M2 macrophages.

LFs and inflammatory effectors, in particular M2 macrophages, are indeed reported as the main cellular effectors in the genesis of the inflammatory‐ and immune‐driven fibrosis of the lung interstitium and airways.^[^ ^31^ ^]^ LFs that might have different origins (as result of EMT, the differentiation of parenchymal stromal stem cells and the migration of circulating fibrocytes into the lung) differentiate into myofibroblasts, which then contribute to extracellular matrix deposition. Alveolar macrophages differentiate toward a reparative profile and, by releasing factors such as TGF‐β, CTGF, and CCL18, favor EMT, fibroblast‐to‐myofibroblast differentiation, and extracellular matrix production.^[^ ^32^, ^33^ ^]^ Thus, LIP‐HA44700‐Im possesses the ability to not only interfere with LFs viability and collagen production but also modulate macrophage activation and proinflammatory cytokines production. Taken together with the evidence of lower uptake and cytotoxicity in CD44 negative epithelial cells, the case for assessing the efficacy of LIP‐HA44700‐Im in a mouse model of postinflammatory fibrosis was very strong.

The activity of the LIP‐HA44700‐Im formulation on a mouse model of bleomycin‐induced pulmonary fibrosis was assessed after local delivery by IT route and was compared with that of the Im free drug, with both being administered either via the IT or IP routes. Our results indicate that the IT administration of LIP‐HA44700‐Im is very effective in preventing lung damage edema and fibrosis. In addition, animals treated with IT LIP‐HA44700‐Im had a significantly lower degree of inflammation, which is demonstrated by the following considerable evidence: 1) significant decrease in neutrophil activity; 2) significant decrease in tissue lymphocyte infiltration; 3) significant decrease in the rate of alveolar inflammation (as demonstrated by decreased neutrophil, lymphocyte, and macrophage counts in BAL); 4) significant increase in tissue expression of IkB‐alpha and significant decrease of tissue NF‐kB and TRAF‐6 expression. The inhibition of local inflammation and neutrophil/lymphocyte/macrophage activation led to a reduction in the expression of MMPs (MMP2 and MMP9), which are crucial players in tissue remodeling and fibrogenesis, as has been clearly demonstrated by the significantly lower degree of collagen deposition that we have previously observed in treated animals.^[^ ^33^, ^34^ ^]^ Moreover, the inhibition of the NF‐kB signaling pathway may provide a number of favorable effects, preventing the action of several pro‐inflammatory cytokines, among all IL‐17 activity on LFs.^[^ ^33^ ^]^ Thus, this considerable amount of evidence clearly demonstrates that local treatment with LIP‐HA44700‐Im prevents pulmonary fibrosis and markedly downregulates local inflammation and MMP expression. This is in line with our previous report in an analogous experimental setting with anti‐CD44Ab‐coated‐Im‐loaded GNPs,^[^ ^9^ ^]^ which efficiently decreased the pathological effects induced by bleomycin treatment, collagen deposition and fibrotic tissue. In addition, although this study has not been designed to address the systemic toxicity of pulmonary LIP‐HA44700‐Im delivery, none of the treated animals showed any sign of systemic toxicity (weight loss, sudden death). Moreover, animals treated with IT LIP‐HA44700‐Im experienced a lower degree of weight loss, lower Ashcroft scores, lower degrees of tissue and BAL inflammation, and lower collagen deposition than in both routes of Im free drug administration. The reasons for LIP‐HA44700‐Im higher efficacy in this animal model are still to be clarified. However, we can hypothesize that encapsulation and targeting may increase and prolong drug effects and partially preserve the exposure of lung epithelial and endothelial cells to a potentially toxic compound.^[^ ^35^, ^36^, ^37^ ^]^ More detailed in vitro and ex vivo molecular studies are, nevertheless, necessary to refine this hypothesis. However, as a conclusion, we would like to highlight the degree of innovation of the approach presented here, which allows, through the local administration of functionalized liposomes, to achieve therapeutic results similar, if not superior to systemic therapy, limiting systemic toxicity and opening the way for further studies in this field.

## 4 Experimental Section

4.1

4.1.1

##### Preparation of Liposomes

Im and all phospholipids were purchased from Merck (Milan, Italy).

The solubility of Im in citrate buffer (0.1 m, pH 4.5) and in ammonium sulphate solution (0.1 m, pH 5.7) was determined at 25 °C by thermodynamic (or equilibrium) solubility assay that investigates the solubility of a compound as a saturated solution in equilibrium. A suspension of Im was prepared in each of the two media; the mixtures were maintained under magnetic stirring over 24 h and then filtered by 0.45 μm (PTFE, purchased by VWR International Srl, Milan, Italy). The citrate and ammonium sulphate filtrates were analyzed by reverse phase‐high‐performance liquid chromatography (RP‐HPLC) method described below to establish Im concentration.

LIP‐Im were prepared by thin lipid hydration method mixing together DPPC, CHOL, and PG in 70:30:3 molar ratio dissolved in chloroform and evaporated by a rotary evaporator. The resulting lipids were dried under vacuum overnight and then were hydrated with citrate buffer (900 μL, pH 4.5), vortexed, and bath sonicated. Next, the obtained suspension was sequentially extruded (Extruder, Lipex, Vancouver, Canada) at 40 °C under nitrogen through 400 and 200 nm polycarbonate filters (Costar, Corning Incorporated, NY). Gel filtration using SepharoseCL‐4B columns (Merck), eluting with HEPES buffer (20 mM, pH 7.4) at room temperature, was used to change the external buffer. To encapsulate Im into liposomes, Im (500 μg/100 μL) in HEPES buffer was added to liposomes and incubated at 37 °C for 30 min. Finally, unencapsulated Im was removed from LIP‐Im by gel filtration, as reported above. LIP (i.e., without adding Im) were also prepared. All liposomes were stored at 4 °C.

##### Preparation of Decorated Liposomes

A conjugate between sodium hyaluronate (HA) (44 700 Da) (Lifecore Biomedical, Chaska, USA) and the 1,2‐dipalmitoyl‐sn‐glycero‐3‐phosphoethanolamine (DPPE) phospholipid (HA‐DPPE) was prepared using the method described by Arpicco et al. to obtain decorated liposomes (LIP‐HA44700‐Im). LIP‐HA44700‐Im were prepared by adding the HA‐DPPE conjugate (3% molar ratio) in citrate buffer (900 μL, pH 4.5) during the hydration of the lipid film composed of DPPC:CHOL in a 70:30 molar ratio. LIP‐HA44700 were also prepared.

For cellular internalization studies, fluorescent LIP (plain and decorated) were prepared using fluorescein or Lissamine [Rhodamine B 1,2‐dihexadecanoyl‐sn‐glycero‐3‐phosphoethanolamine, triethylammonium salt (DHPE‐rhodamine)] (ThermoFisher Scientific, Milan, Italy). In the first case, a 10 mM solution of fluorescein‐5‐(and‐6)‐sulfonic acid trisodium salts in HEPES buffer (900 μL, pH 7.4) was added to hydrate the lipid film. The suspension was then extruded and purified by gel filtration, as previously reported. Rhodamine containing liposomes were prepared by adding in the liposomal formulations DHPE‐rhodamine (1% molar ratio).

##### Liposomes Characterization

The mean particle size and PDI of liposomes were determined at 25 °C using quasielastic light scattering (QELS) on a nanosizer (Nanosizer Nano Z, Malvern INST., Malvern, UK). The selected angle was 173° and the measurement was made after a 1/10 dilution of the liposome suspension in MilliQ water. Each measurement was performed in triplicate. The particle‐surface charge of liposomes was investigated by zeta potential measurements at 25 °C applying the Smoluchowski equation and using the Nanosizer Nano Z. Measurements were carried out in triplicate.

Liposomes morphology was determined by cryo‐TEM analysis. The diluted samples were dropped onto 300 Mesh holey carbon films (Quantifoil R2/1) and quench‐frozen in liquid ethane using a cryo‐plunge workstation (made at LPS Orsay). The specimens were then mounted on a precooled Gatan 62 specimen holder, transferred in the microscope (Phillips CM120) and observed at an accelerating voltage of 120 kV (Centre Technologique des Microstructures, CTμ, platform of the Université Claude Bernard Lyon 1, Villeurbanne, France).

The amount of Im loaded into liposomes was determined by RP‐HPLC. The liposomal formulation was appropriately diluted with acetonitrile, bath sonicated, vortexed, and centrifuged (10 min, 2150 g). The supernatant was filtered (0.45 μm PTFE, purchased from VWR International Srl) and analyzed by HPLC. Analyses were performed on a HP 1200 chromatograph system (Agilent Technologies, Palo Alto, CA, USA) equipped with a quaternary pump (model G1311A), a membrane degasser (G1322A), a multiple wavelength UV detector (MWD, model G1365D), and a fluorescence detector (G1321A) integrated into the HP1200 system. Data analysis was performed using a HP ChemStation system (Agilent Technologies). The HPLC method was adapted from Roth O.^[^ ^38^ ^]^ The sample was eluted on an EC 250/4.6 Nucleosil 100‐5 C18 HD (Macherey‐Nagel, Dueren, Germany). The injection volume was 20 μL (Rheodyne, Cotati, CA, USA). The mobile phase consisted of acetonitrile and potassium dihydrogen phosphate (60 mM, pH 6.5), (60/40, v/v) at a flow‐rate of 1.0 mL min^−1^. The column effluent was monitored at 270 nm and referenced against an 800 nm wavelength. Quantitation of the compound was done by interpolating the peak's area of the compound in a calibration curve obtained by analyzing standard solutions in a concentration range of 10 to 200 μM (*r* ^2^ > 0.999). The phospholipid amount was determined in each liposomal preparation by phosphate assay after destruction with perchloric acid. To evaluate the Im release from liposomes, the formulations were incubated at 37 °C in HEPES buffer or FBS (Merck) for various periods. Drug leakage was determined after the purification of liposomes (200 μL) by chromatography on Sepharose CL‐4B columns, eluting with HEPES buffer. Drug content was then measured by HPLC, as previously described, and compared with initial values. The physical stability of the liposomal formulations under storage conditions (4 °C) was determined by evaluating mean diameter, zeta potential and drug leakage at different time intervals.

##### In Vitro Tests

The A549 (CD44^high^), Calu‐3 (CD44^mid^), and 16HBE (CD44^low^) cell lines (all purchased from ATCC, Manassas, VA, USA) were cultivated in high glucose Dulbecco′s Modified Eagle′s Medium (DMEM, Euroclone, Milan, Italy) supplemented with 10% heat‐inactivated FBS (Euroclone), 100 U mL^−1^ P/S solution (Euroclone) and 100 U mL^−1^ L‐glutamine (Euroclone).

LFs were isolated from BAL of patients affected by BOS and CTD‐ILD, obtained following standard recommendations. 6 × 10^6^ cells were seeded in the same medium of cultivation. Single *foci* of LFs, formed in weeks 1‐3, were isolated and cultivated.

Human monocytic THP‐1 cells were maintained in culture in Roswell Park Memorial Institute medium (RPMI 1640, Euroclone,) containing 10% heat‐inactivated FBS (Euroclone), 100 U mL^−1^ P/S solution (Euroclone) and 100 U mL^−1^ L‐glutamine (Euroclone). THP‐1 monocytes were differentiated into macrophages by 24 h incubation with 5 ng mL^−1^ phorbol 12‐myristate 13‐acetate (PMA, Merck) followed by 24 h incubation in RPMI medium. Macrophages were polarized to M1 macrophages by incubation with IFN‐γ (20 ng mL^−1^, R&D system) and LPS (10 pg mL^−1^, Merck). Macrophage M2 polarization was obtained by incubation with interleukin‐4 (20 ng mL^−1^, R&D Systems, Minneapolis, MN, USA) and interleukin‐13 (20 ng mL^−1^, R&D Systems).

##### Confocal Microscopy

A549 and LFs were seeded in 35 mm glass bottom petri dishes (Corning Costar, Lowell, MA, USA) at a density of 1.5 × 10^4^ cells. After 24 h, cells were incubated with fluorescently labeled LIP and LIP‐HA44700 for 4 h and 24 h at 37 °C. Subsequently, cells were washed with phosphate‐buffered saline (PBS), fixed with 4% paraformaldehyde, and DAPI solution was added to label cell nuclei. Cells were observed by confocal laser microscopy Fluoview FV10i (Olympus, Tokyo, Japan).

##### Flow Cytometry for Liposome Uptake

Calu‐3, 16HBE, macrophages derived from THP‐1, A549, BOS, and CTD‐ILD‐LFs were seeded in 12‐well plates at a density of 2.5 × 10^4^ cells. After 24 h, cells were incubated with fluorescently labeled LIP and LIP‐HA44700 for 4 h at 37 °C. Cells were then washed with PBS, harvested in cytometer tubes, and analyzed using a flow cytometer (Navios, Beckman Coulter, CA, USA) to quantify the fluorescent signal. Fluorescent liposomes were detected using the FL‐1 channel, acquiring 10 000 events per sample, using Kaluza software and CellQuest software for Navios and Becton–Dickinson flow cytometers, respectively. Cells incubated in the absence of liposomes were used as controls.

##### Cell Viability Assay

The MTT test (Merck) was used to assess the viability of LFs. Briefly, 5 × 10^3^ cells were incubated with Im‐loaded liposomes and Im both 30 μM for 2 h at 37 °C. Subsequently, fresh medium was added to continue incubation up to 24, 48, and 72 h. Results are expressed as percentage of variation versus untreated LFs (CTR) set to 100%.

The cell supernatant was collected after centrifugation at 400 × *g* at 4 °C for 5 min and LDH activity was detected using CytoTox 96 Non‐Radioactive Cytotoxicity Assay (Promega, Madison, WI, USA), according to the manufacturer's protocol. Three wells were used per experimental condition during the experiment.

##### IL‐6 Determination

IL‐6 was titled using a commercial kit (Human Immunoassay, R&D Systems), with a quantitative enzyme immunoassay technique, according to the manufacturer's instructions. The results are expressed in pg mL^−1^.

##### Animals

Male CD1 mice (25–30 g, Envigo, Milan, Italy) were accommodated in a controlled location. They received food and water ad libitum. The University of Messina Review Board for animal care (OPBA) approved the study (Ministry of Health, 508/2023‐PR). All in vivo experiments followed the new directives of the USA, Europe, and Italy, as well as the ARRIVE guidelines.

##### Induction of Lung Injury

Bleomycin administration was performed as previously described.^[^ ^39^ ^]^ Bleomycin sulphate (1 mg kg^−1^ body weight) was delivered in a single IT administration. A volume (100 μL) was injected at end‐expiration to guarantee delivery to the distal airways. This was immediately followed by air (300 μL). Treatments were administered everyday starting from the tenth day. Animals were euthanized 28 days after bleomycin injection, and the tissues were harvested for further analysis.

##### Experimental Groups

Mice were randomized into the following experimental groups (*n* = 10):

Sham + vehicle group: Identical to the bleomycin + vehicle group, but animals received IT instillation of saline (0.9% w/v) instead of bleomycin and were treated daily with the vehicle (saline);

Bleomycin + vehicle: Mice received bleomycin administration and were treated daily with the vehicle (saline) (50 μL);

Bleomycin + HEPES: Mice received bleomycin administration and were treated intratracheally with HEPES (50 μL);

Bleomycin + Im IT: Mice received bleomycin administration and were treated intratracheally with Im (Im‐IT) (50 μL of 150 μg mL^−1^);

Bleomycin + Im IP: Mice received bleomycin administration and were treated daily with Im (Im‐IP) (10 mg mL^−1^);

Bleomycin + LIP‐HA44700‐Im: Mice received bleomycin administration and were treated daily with LIP‐Im and LIP‐HA44700‐Im (50 μL of 150 μg mL^−1^).

Body weight was assessed daily up to 28 days.

Mice were euthanized 28 days after bleomycin installation and the tissues were harvested for analyses of injury and inflammation.

##### Animal Bronchoalveolar Lavage

Twenty‐eight days after bleomycin installation, mice were euthanized, and the tracheas were cannulated to perform the lavage, as previously described.^[^ ^40^ ^]^ In total, 0.5 mL PBS (GIBCO, Paisley, UK) was used. The BAL fluid recovered was spun, the pelleted cells were collected, and the supernatants were stored at −20 °C. In the presence of trypan blue, total BAL cells were counted using a hemocytometer. The total leukocyte number was determined, in duplicate, using a hemocytometer (in a Burker chamber). For differential white blood counts, a smear was prepared from the cell pellet in BALf and stained with Wright–Giemsa. After staining, the differential count was carried out following the standard morphological protocol under a light microscope.

##### Histological Examination

Lung tissue samples were collected 28 days from bleomycin injection. After fixing the tissues in buffered formaldehyde solution (10% in PBS), histological sections were stained with haematoxylin and eosin, and evaluated using a Leica DM6 (Milan, Italy) microscope.^[^ ^41^ ^]^ The severity of lung fibrosis was scored on a scale from 0 to 8, as already published.^[^ ^40^ ^]^ Lung sections were stained with Masson's trichrome for fibrosis.

##### MPO Assay

MPO colorimetric activity assay was determined as previously described.^[^ ^41^ ^]^ It was defined as the quantity of enzyme that degraded 1 μmol of peroxide per min at 37 °C and is expressed in units per gram of wet‐tissue weight.

##### Western Blot Analyses

After treating LFs with LIP‐Im, LIP‐HA44700‐Im and 30 μM Im for 24 h, cells were lysed with lysis buffer (50 mM Tris‐HCl [pH 7.4], 150 mM NaCl, 10% glycerol, 1% NP‐40), protease inhibitor cocktail (Merck) and phosphatase inhibitor (Roche), gently vortexed for 20 min at 4 °C and centrifuged for 15 min at 13 200 rpm at 4 °C. Supernatants were quantified using the Pierce BCA Protein Assay Kit (Thermo Fisher Scientific). Proteins from cell extracts (20 μg) were loaded and separated in 8% SDS‐PAGE. After electrophoresis, the gels were transferred to polyvinylidene difluoride membranes (Millipore), and then blocked (5% no fat milk in 0.1% Tween 20 TBS) and incubated with the primary Ab (1:1000 in TBST + 2% BSA; overnight at 4 °C or 2 h at room temperature): anti‐c‐Abl (phospho Y412) (ab4717, Abcam), anti‐Col1a1 (ab34710, Abcam), and anti‐β‐Actin (MAB1501R, Chemicon). After washing, the membranes were incubated with the appropriate HRP‐conjugated secondary Ab (1:5000 in TBST + 2% BSA; 2 h at room temperature; anti‐mouse A4416 and anti‐rabbit A0545, Merck). Immunoreactivity was detected using ECL reagents (Amersham), and acquired with the ChemiDoc imaging system (Image Lab, Bio‐Rad, Segrate, Italy).

For animal models, western blots were performed as described in our previous studies.^[^ ^42^ ^]^ Briefly, lung tissues from each mouse were suspended in an extraction buffer containing pepstatin A (0.15 μM), phenylmethylsulfonyl fluoride (0.2 mM, PMSF), sodium orthovanadate (1 mM), and leupeptin (20 μM); they were then homogenized at the highest setting for 2 min and centrifuged at 1000 g for 10 min at 4 °C. Supernatants contained the cytosolic fractions, while the pellets represent the nuclear ones. Pellets were resuspended in a second buffer containing sodium chloride (150 mM, NaCl), 1% Triton X‐100, ethylene glycol tetraacetic acid (1 mM, EGTA), tris‐chloridric acid (10 mM, HCl) pH 7.4, PMSF (0.2 mM), ethylenediaminetetraacetic acid (1 mM, EDTA), sodium orthovanadate (0.2 mM), and leupeptin (20 μm). After centrifugation at 4 °C and 15 000 g for 30 min, the nuclear proteins containing the supernatants were stored at −80 °C for further analysis. Specific primary antibody: anti‐IkB (1:1000, Santa Cruz Biotechnology, Dallas, TX, USA) or anti‐NF‐kB p65 (1:1000; Santa Cruz Biotechnology) or anti‐p‐p38 (1:1000, Santa Cruz Biotechnology) or anti‐p‐JNK (1:1000, Santa Cruz Biotechnology) or anti‐MMP2 (1:1000, Santa Cruz Biotechnology) or anti‐MMP9 (1:1000, Santa Cruz Biotechnology) or anti‐TRAF‐6 (1:1000, Santa Cruz Biotechnology) were mixed in 1×PBS, nonfat dried milk (5% w/v), and Tween‐20 (0.1%), and incubated at 4 °C overnight. Subsequently, blots were incubated with peroxidase‐conjugated bovine antimouse IgG secondary antibody or peroxidase‐conjugated goat antirabbit IgG (1:2000, Jackson Immuno Research, PA, USA) for 1 h at room temperature.^[^ ^43^ ^]^ To verify that membranes were loaded with equal amounts of protein, they were also incubated with the antibody against laminin (1:1000; Santa Cruz Biotechnology) and β‐actin (1:1000; Santa Cruz Biotechnology). Signals were detected with enhanced chemiluminescence detection system reagent, according to the manufacturer's instructions (Super‐Signal West Pico Chemiluminescent Substrate, Pierce). The relative expression of the protein bands was quantified by densitometry using Bio‐Rad ChemiDoc XRS software and standardized to b‐actin levels. Images of blot signals (8‐bit/600‐dpi) were imported to analysis software (Image Quant TL, v2003).

##### Immunohistochemical Localization

The immunohistochemical techniques used have been previously described.^[^ ^44^ ^]^ The antibodies that were incubated O/N on the brain sections were anti‐TGF‐b (Millipore, 1:500 in PBS, v/v, AB152, Burlington, MA, USA), anti‐CD4 (Santa Cruz Biotechnology, 1:300 in PBS, v/v, 65G10 sc‐32258, Dallas, TX, USA), and anti‐CD8 (Santa Cruz Biotechnology, 1:50 in PBS, v/v, LB509 sc‐58480, Dallas, TX, USA). To verify the specificity of the antibodies, sections of five mice for each group were treated either with a primary, or only with a secondary antibody. Images were taken using a Leica DM6 microscope, and the ImageJ IHC profiler plug‐in was used for densitometric analysis. When this is selected, a histogram profile of the deconstructed DAB image is automatically traced and a corresponding score log is shown.^[^ ^45^ ^]^ The histogram profile corresponds to the positive pixel intensity value obtained from the computer program.^[^ ^46^ ^]^ Immunohistochemical analysis was performed by experienced scientists who were unaware of the treatment performed.

##### In Vitro Statistical Analysis

Prior to analysis, data were examined for normality using the Shapiro–Wilk test. If necessary, data were log‐transformed to meet normality assumptions. Data are presented as mean ± standard deviation (SD), unless otherwise stated. Sample size (*n*) for each experiment is indicated in the corresponding figure legend. Statistical comparisons between groups were conducted using Student's *t*‐test for two‐group comparisons or one‐way ANOVA followed by Tukey's post hoc test for multiple comparisons. A significance level of *α* = 0.05 was set for all analyses. All statistical analyses were performed using GraphPad Prism 8.

##### In Vivo Statistical Analyses

All values in the figures and text are expressed as the mean ± SEM of *n* = 10 number of animals. The results were analyzed by one‐ or two‐way ANOVA followed by a Bonferroni post hoc or Dunnett post hoc test for multiple comparisons. A *p*‐value < 0.05 was considered significant. *p < 0.05 versus sham, #*p* < 0.05 versus vehicle, **p < 0.01 versus sham, ##*p* < 0.01 versus vehicle, ***p < 0.001 versus sham, ###*p* < 0.001 versus vehicle, °*p* < 0.05 versus Im‐IT and Im‐IP, °°*p* < 0.01 versus Im‐IT and Im‐IP, °°°*p* < 0.001 versus Im‐IT and Im‐IP.

## Conflict of Interest

The authors declare no conflict of interest.

## Author Contributions

**Sara Bozzini**: writing—review and editing, writing—original draft, validation, methodology, investigation, formal analysis, data curation. **Valeria Bincoletto**: writing—review and editing, writing—original draft, validation, methodology, investigation, formal analysis, data curation. **Laura Pandolfi**: writing—review and editing, validation, methodology, investigation, formal analysis, data curation. **Roberta Fusco**: writing—review and editing, validation, methodology, investigation, formal analysis, data curation. **Rosanna Di Paola**: writing—review and editing, methodology, investigation, data curation, supervision, conceptualization. **Salvatore Cuzzocrea**: writing—review and editing, methodology, investigation, data curation, supervision, conceptualization. **Ilaria Andreana**: writing—review and editing, methodology, investigation, software, data curation. **Barbara Rolando**: writing—review and editing, methodology, investigation, data curation. **Eleonora Bozza**: writing—review and editing, methodology, investigation, data curation. **Cecilia Bagnera**: writing—review and editing, methodology, investigation, data curation. **Manuela Monti**: writing—review and editing, methodology, investigation, data curation. **Barbara Stella**: writing—review and editing, conceptualization. **Federica Meloni**: writing—review and editing, writing—original draft, supervision, funding acquisition, project administration, resources, conceptualization**. Silvia Arpicco**: writing—review and editing, writing—original draft, supervision, funding acquisition, project administration, resources, conceptualization. **Sara Bozzini** and **Valeria Bincoletto** contributed equally to this work.

## Supporting information

Supplementary Material

## Data Availability

The data that support the findings of this study are available from the corresponding author upon reasonable request.
