# Supplementary material for: Hyaluronic Acid‐Decorated Liposomes for the Intrapulmonary Delivery of Imatinib: A Targeted Treatment for Postinflammatory Pulmonary Fibrosis
Source: Small Sci. 2025 Jun 10;5(8):2500144. doi: 10.1002/smsc.202500144 (PMC12362731; doi:10.1002/smsc.202500144)
Supplement: Supplementary file 1 — Supplementary Material [file SMSC-5-2500144-s001.pdf]

Supporting Information

**Hyaluronic acid-decorated liposomes for the intrapulmonary delivery of imatinib: a targeted treatment for post-inflammatory pulmonary fibrosis**

*Sara Bozzini, Valeria Bincoletto, Laura Pandolfi, Roberta Fusco, Rosanna Di Paola, Salvatore Cuzzocrea, Ilaria Andreana, Barbara Rolando, Eleonora Bozza, Cecilia Bagnera, Manuela Monti, Barbara Stella, Federica Meloni *, Silvia Arpicco **

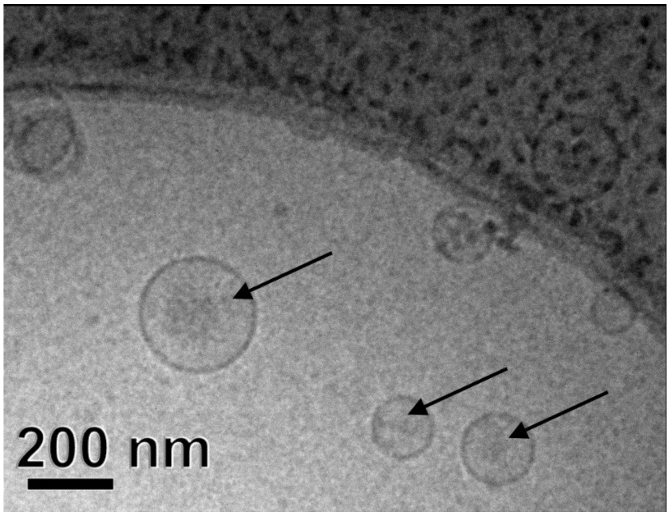


**Figure S1.** Representative Cryo-TEM image of LIP-Im. The arrows indicate Im precipitated inside the liposome structure.
